# Spotlight on Ferroptosis: Iron-Dependent Cell Death in Alzheimer’s Disease

**DOI:** 10.3389/fnagi.2020.00196

**Published:** 2020-07-14

**Authors:** Azhaar Ashraf, Po-Wah So

**Affiliations:** Department of Neuroimaging, Institute of Psychiatry, Psychology, and Neuroscience, King’s College London, London, United Kingdom

**Keywords:** Alzheimer’s disease, cystine/glutamate antiporter, ferroptosis, glutathione peroxidase-4, iron, lipid peroxidation

## Abstract

Alzheimer’s disease is an emerging global epidemic that is becoming increasingly unsustainable. Most of the clinical trials have been centered around targeting β-amyloid and have met with limited success. There is a great impetus to identify alternative drug targets. Iron appears to be the common theme prevalent across neurodegenerative diseases. Iron has been shown to promote aggregation and pathogenicity of the characteristic aberrant proteins, β-amyloid, tau, α-synuclein, and TDP43, in these diseases. Further support for the involvement of iron in pathogenesis is provided by the recent discovery of a new form of cell death, ferroptosis. Arising from iron-dependent lipid peroxidation, ferroptosis is augmented in conditions of cysteine deficiency and glutathione peroxidase-4 inactivation. Here, we review clinical trials that provide the rationale for targeting ferroptosis to delay the pathogenesis of Alzheimer’s disease (AD), potentially of relevance to other neurodegenerative diseases.

## Introduction

Alzheimer’s disease (AD) is increasing at an alarming rate and is an emerging epidemic. Current incidence world-wide is ~50 million and expected to triple by 2050. The economic costs of AD, the most common cause of dementia, are $260 billion and projected to reach $1167 billion by 2050 (Alzheimer’s Disease International, 2019). Thus, there is a great impetus to develop treatments to delay AD pathogenesis.

AD is classically defined by β-amyloid plaques and neurofibrillary tangles. Many clinical trials directed at lowering β-amyloid have met with limited success. One reason for the failures could be that 50% of AD patients exhibit α-synuclein pathology, while 30% show TDP43 pathology (Robinson et al., 2018). We suggest sole targeting of β-amyloid or tau may not be fruitful in AD as contributions from α-synuclein and TDP43 significantly modify disease pathogenesis—alternative disease-modifying targets are crucial.

Iron dyshomeostasis appears to be a common theme, unifying neurodegenerative diseases including AD, Lewy Body disease, and frontotemporal dementia (FTD). Iron has been shown to promote the aggregation and pathogenicity of β-amyloid (Rottkamp et al., 2001), tau (Sayre et al., 2000), α-synuclein (Xiao et al., 2018) and indirectly, TDP43 (Jeong et al., 2009; Joppe et al., 2019). We hypothesize that iron dyshomeostasis is widespread in neurodegenerative diseases and ensuing mechanisms underlying iron toxicity may provide novel disease targets. This unified and unique approach—targeting iron dyshomeostasis—is likely to benefit dementia patients, the majority of which are affected by AD, but also orphan diseases e.g., FTD. Here, we review the evidence from clinical trials concerning the rationale of targeting ferroptosis for AD.

## Importance of Iron Homeostasis

Iron is an essential metal for neurons, required for mitochondria respiration as well as other processes e.g., myelination and neurotransmitter synthesis (Ward et al., 2014; Ashraf et al., 2018). Iron can exist in oxidized and reduced states, ferric iron (Fe^3+^), and ferrous iron (Fe^2+^), respectively. Ferrous iron predominantly contributes to the cellular labile iron pool (LIP), participating in key metabolic reactions but also toxic reactions that can lead to oxidative stress and eventually cell death. Excessive build-up of LIP is avoided by sequestration of iron, in a bioavailable yet non-toxic form, by ferritin (Harrison and Arosio, 1996). Ferritin works in concert with ferroportin, the only known cellular iron exporter, aided by the ferroxidase, ceruloplasmin, to regulate LIP (De Domenico et al., 2007). Tight regulation of iron metabolism is pivotal to ensure neuronal homeostasis—both iron excess and deficiency are associated with neurodegeneration (Goodman, 1953; Youdim, 2008; Ashraf et al., 2019).

## Labile Iron Pool and Its Role in Ferroptosis

Increased LIP can lead to the generation of reactive oxygen species *via* Fenton reaction, eventually resulting in catastrophic membrane rupture (Kakhlon and Cabantchik, 2002; Petrat et al., 2002; Kruszewski, 2003). Coincident depletion of glutathione (GSH) or inactivation of glutathione peroxidase-4 (GPX4) leads to a newly characterized form of cell death, coined ferroptosis (Dixon et al., 2012). Neuron-specific GPX4 depletion has been shown to lead to neurodegeneration, highlighting this pathway as a future therapeutic target (Seiler et al., 2008). GPX4 is a master regulator of ferroptosis by functioning as a lipid repair enzyme and detoxifying lipid hydroperoxides, utilizing GSH as an essential cofactor. GSH synthesis/levels are reliant on cellular cysteine availability. Cysteine is imported into cells in its oxidized form, cystine, in exchange for glutamate by the cystine/glutamate antiporter (system ${\text{X}}_{\text{c}}^{-}$). Increased extracellular glutamate in concert with glutaminolysis may contribute to detrimental excitotoxicity (Dixon et al., 2012; Gao et al., 2015; Stockwell et al., 2017; Ashraf et al., 2020), and possibly a feature of ferroptosis.

System ${\text{X}}_{\text{c}}^{-}$-inhibition attenuates GSH levels, inactivates GPX4, and enhances lipid peroxidation (Dixon et al., 2012). Polyunsaturated fatty acids (PUFAs), especially arachidonic acid, in membrane lipids are preferentially oxidized during ferroptosis. Arachidonic acid is activated by Acyl-CoA Synthetase Long-Chain Family Member 4 (ACSL4) for incorporation into phosphatidylethanolamines (and membranes). Oxidized phosphatidylethanolamines are proximate executors of ferroptosis and hence ACSL4 expression modulates ferroptosis susceptibility (Doll et al., 2017). Notably, long-term use of pioglitazone, recently characterized to be an ACSL4 inhibitor, is associated with attenuated risk of dementia in type 2 diabetes mellitus patients (Heneka et al., 2015).

While iron enhances lipid peroxidation *via* the non-enzymatic Fenton reaction, lipid peroxidation can also be catalyzed by specific non-heme, iron-containing lipoxygenases, such lipoxygenases also confer vulnerability to ferroptosis (Yang et al., 2016). GPX4-ablation in mice and cells revealed downstream 12/15-lipoxygenase-derived lipid peroxidation, trigger apoptosis-inducing factor-mediated cell death, and subsequent oxidative stress (Seiler et al., 2008). Moreover, neuron-specific ablation of GPX4 in the forebrain (cerebral cortex and hippocampus) was associated with an increase in markers associated with ferroptosis including increased lipid peroxidation, extracellular-regulated kinase (ERK) 4 and neuroinflammation (Hambright et al., 2017). The susceptibility of AD vulnerable neuronal populations to ferroptosis is suggestive of its role in AD.

During ferroptosis, cytosolic ferritin may undergo lysosomal breakdown (ferritinophagy) to further contribute to LIP—ferritinophagy appears to augment cysteine deficiency-induced ferroptosis (Gao et al., 2016; Hou et al., 2016). While total cellular iron levels may be unchanged, an augmented LIP renders cells more susceptible to ferroptosis. The increased influx of iron into the mitochondria induces the accumulation of reactive oxygen species and lipid peroxidation. Lipid peroxidation is enhanced in cysteine deprivation *via* hyperpolarized mitochondrial membrane potential (Gao et al., 2019). The distinguishing features of ferroptosis are evident cytologically, in the form of condensed mitochondrial membrane and mitochondrial volume shrinkage (Yagoda et al., 2007; Stockwell et al., 2017). Interestingly, genetic factors including TDP43, amyloid precursor protein (APP), APOE may play a pivotal role in modifying mitochondrial functionality. Suppressing localization of TDP43 in the mitochondria inhibited TDP43-mediated neurotoxicity (Wang et al., 2016). Electron microscopic analysis of FTD and amyotrophic lateral sclerosis patients with TDP43 pathology revealed prominent mitochondrial impairment, including abnormal and/or depleted cristae, concordant with ultrastructural changes observed in both cellular and animal models of TDP43 proteinopathy (Wang et al., 2019). Mechanistically, TDP43 expression attenuated mitochondrial membrane potential, suppressed mitochondrial complex I activity, and impaired mitochondrial ATP synthesis. Moreover, downregulation of LonP1 (mitochondrial protease) augmented TDP43 levels which exacerbated TDP43-induced mitochondrial damage and neurodegeneration (Wang et al., 2019).

Mitochondria isolated from AD brains show increased accumulation of APP and β-amyloid associated with reduced ability of mitochondria to import nuclear-encoded proteins and impaired cytochrome c oxidase activity (Devi et al., 2006; Hansson Petersen et al., 2008). Tau mutant mice and triple transgenic mice harboring APP and tau mutations demonstrated impaired mitochondrial respiration, increased production of reactive oxygen species, and augmented oxidative stress (David et al., 2005; Rhein et al., 2009; Yao et al., 2009). APOEε4 genotype is a major susceptibility risk locus particularly in AD, associated with enhanced mitochondrial fusion and decreased fission (Simonovitch et al., 2019). APOE4 has been found to negatively modify effects of iron on brain functionality before the manifestation of cognitive impairment (Kagerer et al., 2020), and can regulate iron-homeostatic proteins like ferritin to increase an individual’s risk of conversion to AD (Ayton et al., 2015). Combining the different lines of evidence, a pivotal involvement of proteinopathies is indicated in inducing iron dyshomeostasis, lipid peroxidation, and mitochondrial damage which are reminiscent of changes consistent with ferroptosis. This proposition awaits experimental validation to elucidate a direct role of the misfolded proteins in ferroptosis in the context of neurodegenerative diseases.

## Evidence for Ferroptosis in AD

### Iron Chelators

A 2-year Phase II clinical trial reported desferrioxamine, an iron chelator, attenuates cognitive decline in AD (Crapper McLachlan et al., 1991). However, desferrioxamine treatment was not further pursued owing to its lack of blood-brain-barrier (BBB) penetrance. Intranasal deferoxamine overcomes this problem and shown to improve cognition in a mouse AD model (Fine et al., 2012, 2015). Iron chelation attenuated oxidative stress, lowered β-amyloid load, and tau hyperphosphorylation (by inhibition of cyclin-dependent kinase-5 and glycogen synthase kinase activity; Guo et al., 2013a; Guo et al., 2013b).

Deferiprone is an orally active, brain penetrant iron-chelator, approved for use in β-thalassemia, currently, undergoing a Phase II clinical trial in mild cognitive impairment (MCI) and AD (Deferiprone to Delay Dementia—clinicaltrials.gov identifier: NCT03235686; Nikseresht et al., 2019). This trial was preceded by Phase II clinical trials on Parkinson’s disease (PD) which showed reduced brain iron assessed by magnetic resonance imaging (MRI) and cerebrospinal fluid (CSF) ferritin and concomitant ameliorated motor deficits (Table 1, Devos et al., 2014; Martin-Bastida et al., 2017).

**Table 1:** Clinical trials involving iron-chelators in Alzheimer’s disease (AD) and Parkinson’s disease (PD).

Study	Study population	Treatment	Dose	Duration (years)	Outcome measures	Results
Crapper McLachlan et al. (1991)	48 AD cases	Iron-chelator Desferrioxamine	125 mg twice daily for 5 days/week Intramuscular	2	Videorecorder home-behavioral assessment for activities of daily living Wechsler Adult Intelligence Scale-revised Wechsler Memory Scale form 1 Western Aphasia Battery	Significant reduction in rate of decline in activities of daily living (*p* = 0.03). Subjects suffered appetite loss (4) or had gradual weight loss (1).
Devos et al. (2014)	40 PD cases	Iron-chelator Deferiprone	15 mg/kg twice daily Oral	2	Movement Disorders Society—Unified Parkinson’s Disease Rating Scale (MDS-UPDRS) T2* MRI (surrogate measure for iron) Serum iron, ferritin, transferrin and ceruloplasmin CSF iron and ferritin Plasma and CSF oxidative stress markers including malonaldehyde, 8-oxo-7,8-dihydro-2^′^-deoxyguanosine and carbonylated proteins Plasma and CSF antioxidant markers including glutathione peroxidase and superoxide dismutase	Improved motor performance (*p* = 0.002). Reduced iron in substantia nigra (*p* = 0.001). Reduced CSF and blood ferritin, and oxidative stress (*p* < 0.05). Improved CSF antioxidant levels (*p* < 0.05). Subjects suffered agranulocytosis (1) and neutropenia (2).
Martin-Bastida et al. (2017)	22 PD cases	Iron-chelator Deferiprone	10 or 15 mg/kg twice daily Oral	0.5	MDS-UPDRS Mini-mental State of Folstein (cognitive function) Montgomery Asberg dementia rating scale Parkinson’s disease questionnaire-39 (quality of life) T2* MRI Serum iron, hemoglobin and transferrin Plasma ferritin, interleukin-6 tumor necrosis factor alpha	A trend of improved MDS-UPDRS score, indicative of improved motor performance. Reduced dentate and caudate nucleus iron content (*p* < 0.001). Subjects reported exacerbation of pre-existing muscular/joint pain (7), mild gastro-intestinal upset (3), neutropenia and were withdrawn from the study (2) and had increased liver enzymes (1).

Although chelating the LIP in the brain is a tempting strategy, many challenges warrant mention. Since iron is an essential cofactor in multi-fold cellular processes, iron-chelation can have off-target effects and potentially cause untoward effects. The most frequent side-effects are gastrointestinal discomfort including nausea, abdominal pain, vomiting, and diarrhea, which range from mild to moderate (Borgna-Pignatti and Marsella, 2015). The most severe adverse effect experienced by patients on iron-chelator therapy is neutropenia (8.5%) and agranulocytosis (0.9%; Borgna-Pignatti and Marsella, 2015). Regular weekly monitoring of blood counts (especially of white blood cells) in patients taking deferiprone is essential to monitor side-effects particularly neutropenia and agranulocytosis, and the dosage titrated accordingly. Moreover, periodic hepatic and renal functions should be evaluated, as these organs are major sites of iron metabolism.

### Antioxidants

#### Vitamin E

Vitamin E is the most potent biological lipophilic chain-breaking antioxidant (Stocker, 2007), actually comprising α-, β-, γ-, and δ-tocopherols and α-, β-, γ-, and δ-tocotrienols. All react with free radicals to yield a non-radical product and a vitamin E radical with a delocalized and stabilized unpaired electron. The latter then reacts with another free radical or is regenerated by vitamin C (Maguire et al., 1989). Vitamin E neutralizes peroxyl radicals and terminates lipid peroxidation, especially of PUFAs (Brigelius-Flohé, 2009). PUFAs are particularly susceptible to peroxidation due to their high degree of unsaturation and are greatly enriched (25–30% of total fatty acids; Joffre et al., 2019) in brain cell membranes. Long-term PUFA-supplementation during midlife is associated with decreased AD risk in pre-symptomatic (Laitinen et al., 2006; Yassine et al., 2017), although a shorter duration study reported no benefits (Andrieu et al., 2017). By protecting cellular membranes against lipid peroxidation, vitamin E can be considered a disruptor of ferroptosis.

Vitamin E in plasma, serum, and CSF are reduced in AD (de Wilde et al., 2017). Vitamin E (α-tocopherol) supplementation delayed functional decline and reduced caregiver burden in mild to moderate AD (Table 2, Sano et al., 1997; Dysken et al., 2014). Epidemiological studies using older cohorts from the Netherlands and the Cache County (Utah, USA) concluded vitamin E intake is associated with a lower risk of developing AD (Engelhart et al., 2002; Zandi et al., 2004). A Rotterdam study and Canadian health and aging (1991–2002) study reported attenuated risk of cognitive decline in AD patients on high vitamin E supplementation (Devore et al., 2010; Basambombo et al., 2017). Conversely, numerous studies have reported vitamin E does not reduce AD risk or slow AD pathogenesis (Masaki et al., 2000; Luchsinger et al., 2003; Petersen et al., 2005; Gray et al., 2008; Kryscio et al., 2017).

**Table 2:** Clinical trials involving Vitamin E in Alzheimer’s disease (AD), amnestic mild-cognitive impairment (MCI) and cognitively normal.

Study	Study population	Treatment	Dose	Duration (years)	Outcome measures	Results
Sano et al. (1997)	341 AD cases	Vitamin E (α-tocopherol)	2000 IU daily Oral	2	Alzheimer’s Disease Cooperative Study/Activities of Daily Living (ADCS); Mini-mental state examination (MMSE) Blessed-Dementia scale	Delayed time to occurrence of clinical outcomes that reflect substantial functional deterioration (*p* = 0.001). Patients suffered a fall (12); had syncope (6) and had a dental event (1).
Petersen et al. (2005)	769 Amnestic MCI cases	Vitamin E	2000 IU daily Oral	3	Conversion to AD MMSE Alzheimer Disease Assessment Scale–Cognitive Subscale (ADAS-cog) ADCS Clinical dementia rating (CDR) Global Deterioration Scale (GDS) Neuropsychological battery tests	No clinical benefit.
Lloret et al. (2009)	33 AD cases	Vitamin E (α-tocopherol)	800 IU daily Oral	0.5	MMSE Blessed-Dementia Scale Clock drawing test Blood total glutathione levels and oxidized glutathione Blood malondialdehyde	Vitamin E responders showed significantly decreased oxidative stress and improved cognitive performance (*p* < 0.05). Non-responders to Vitamin E showed significant decline in cognition (*p* < 0.02).
Dysken et al. (2014)	613 AD cases	Vitamin E (α-tocopherol)	2000 IU daily Oral	2	ADCS MMSE ADAS-cog Caregiver Activity Survey	Slowed functional decline (*p* = 0.03).
Kryscio et al. (2017)	7,540 cognitively normal	Vitamin E	400 IU daily Oral	6	Memory Impairment Screen Consortium to Establish a Registry in Alzheimer’s Disease	No significant cognitive benefits.

Why has vitamin E shown mixed results in clinical trials? While vitamin E supplementation could simply restore vitamin levels in AD, baseline vitamin E levels are often not checked. Also, vitamin E bioavailability can be variable due to differential gut absorption from competing nutrients e.g., plant sterols; variable brain levels arising from different vitamin E forms with varying pharmacokinetics and circulating high-density lipoproteins levels, the latter transports and mediates brain import of vitamin E. Differential responses of AD patients to vitamin E supplementation has led to stratification of individuals into respondents and non-respondents in one study (Lloret et al., 2009). Vitamin E effectively lowered oxidative stress and maintained cognitive status in respondents. Surprisingly, non-respondents experienced a sharp decline in cognition, to levels even lower than patients receiving a placebo (Lloret et al., 2009). Further investigations are required to successfully exploit vitamin E supplementation.

#### N-Acetylcysteine

GSH is diminished in the hippocampus and frontal cortex in AD (Mandal et al., 2015), while lower plasma GSH is associated with severe cognitive impairment (McCaddon et al., 2003). Restoration of brain GSH with oral supplementation is ineffective as GSH rapidly hydrolyzes and insufficiently BBB-penetrant (Witschi et al., 1992). Similarly, L-cysteine (rate-limiting substrate for GSH synthesis) is inadequate due to extensive metabolism (Borgström and Kågedal, 1990). However, N-acetylcysteine, a precursor of L-cysteine, can efficaciously cross the BBB into the brain (Tardiolo et al., 2018). N-acetylcysteine mediates restoration of brain GSH levels and GPX4 activity in an AD mouse model, counteracting lipid peroxidation (Pocernich et al., 2000; Fu et al., 2006; Hsiao et al., 2012). A small trial showed 6-months N-acetylcysteine treatment did not improve Mini-Mental State Examination (MMSE) scores or daily living (Adair et al., 2001). Midpoint evaluation did show a trend towards a beneficial effect on the MMSE score though (*p* = 0.056), particularly on the letter fluency task (Table 3). N-acetylcysteine therapy may be more robust by increasing bioavailability, e.g., by using N-acetylcysteine amide (Hara et al., 2017). This N-acetylcysteine derivative has augmented permeability through cellular and mitochondrial membranes, as shown in the PD mouse model (Bahat-Stroomza et al., 2005). Interestingly, MCI and AD subjects supplemented with combined N-acetylcysteine, α-tocopherol, folate, vitamin B12, methionine, acetyl-L-carnitine demonstrated either stable or improved cognitive performance and mood/behavior (Remington et al., 2015a,b, 2016).

**Table 3:** Clinical trials involving N-acetylcysteine in Alzheimer’s disease (AD) and mild-cognitive impairment (MCI).

Study	Study population	Treatment	Dose	Duration (years)	Outcome measures	Results
Adair et al. (2001)	47 AD cases	N-Acetylcysteine (NAC)	50 mg/kg daily Oral	0.5	Mini-mental state examination (MMSE) Activities of daily living (ADL) Boston naming test Gesture to command Wechsler memory scale figure reproduction (immediate) Hopkins verbal learning test recall (immediate) and recognition Letter and category fluency Judgement of line orientation	Significantly improved performance on letter fluency (*p* = 0.008). A trend of improved MMSE (*p* = 0.056).
Remington et al. (2015a)	106 AD cases	NAC Folate Alpha-tocopherol B12 S-Adenosyl methionine (SAM) Acetyl-L-carnitine	600 mg 400 μg 30 IU 6 μg 400 mg 500 mg, twice daily Oral	1	Clox-1 and the age- and education-adjusted (AEMSS) Dementia Rating Scale (DRS) Behavioral and psychological symptoms of dementia (BPSD) Neuropsychiatry inventory (NPI) ADL	Improved cognitive performance (*p* < 0.008).
Remington et al. (2015b)	34 MCI cases	NAC Folate Alpha-tocopherol B12 SAM Acetyl-L-carnitine	600 mg 400 μg 30 IU 6 μg 400 mg 500 mg, twice daily Oral	1	AEMSS Dementia Rating Scale (DRS)	Improved cognitive performance (*p* < 0.05).
Remington et al. (2016)	24 AD cases	NAC Folate Alpha-tocopherol B12 SAM Acetyl-L-carnitine	600 mg 400 μg 30 IU 6 μg 400 mg 500 mg, twice daily Oral	1	AEMSS DRS BPSD NPI ADL	No significant improvements or decline observed.

#### Selenium

Selenium is decreased in the hippocampal, temporal, and cortical regions in AD, consistent with attenuated antioxidant capacity and augmented oxidative stress (Varikasuvu et al., 2019). Selenium, as selenocysteine, is essential for GPX4 synthesis. Six-months of consumption of Brazil nuts (high selenium) by MCI subjects replenished selenium levels (Table 4), improving verbal fluency and constructional praxis (Cardoso et al., 2016). Sodium selenate (over 24-weeks) lessened brain deterioration as assessed by diffusion tensor MRI, but did not impact cognitive performance (Malpas et al., 2016). Interestingly, on stratification into responders and non-responders based on the elevation of CSF levels, MMSE scores did not deteriorate in responders compared to non-responders (Cardoso et al., 2019). Conversely, in the PREADVISE clinical trial, selenomethionine did not reduce the incidence of dementia in cognitively healthy males, aged >60 years (Kryscio et al., 2017), but subjects were not stratified according to their CSF selenium status. Furthermore, the incidence of dementia on follow-up was low, possibly attributable to selection bias as participants were more educated than the general population and exhibited greater cognitive reserve. The absence of biomarkers for target engagement of supplements renders the translation of basic scientific findings into robust prevention trials difficult.

**Table 4:** Clinical trials involving Selenium in Alzheimer’s disease (AD) and mild-cognitive impairment (MCI).

Study	Study population	Treatment	Dose	Duration (years)	Outcome measures	Results
Cardoso et al. (2016)	31 MCI cases	Selenium (from Brazilian nuts)	288.75 μg daily Oral	0.5	Consortium to Establish a Registry in Alzheimer’s Disease Selenium status Erythrocyte glutathione peroxidase (GPX4) activity Oxygen radical absorbance capacity (ORAC) Plasma malondialdehyde	Improved verbal fluency (*p* = 0.007) and constructional praxis (*p* = 0.03). Increased blood selenium (*p* < 0.001) and GPX4 (*p* = 0.006).
Malpas et al. (2016)	40 AD cases	Selenate (sodium salt)	30 mg daily Oral	2	Alzheimer’s Disease Assessment Scale cognitive subscale (ADAS-Cog) Mini-mental state examination (MMSE) Controlled oral word association test (COWAT) Category fluency test (CFT) Cogstate computerized battery Structural and diffusion-weighted MRI FDG-PET (glucose metabolism) Biomarker analysis (β-amyloid, total and phosphorylated tau)	Less deterioration observed on the diffusion-weighted MRI (*p* < 0.05). Mild pre-syncope (1) or dropped out of the study due to skin rash and nail changes (2).
Cardoso et al. (2019)	40 AD cases	Selenate (sodium salt)	30 mg daily Oral	0.5	ADAS-Cog MMSE COWAT CFT Cogstate computerized battery Total selenium serum and CSF concentrations	Responders to treatment showed increased serum (*p* = 0.007) and CSF selenium (*p* = 0.03), and showed no significant cognitive decline.

## Targeting Ferroptosis—the Future of AD?

Despite rigorous clinical testing of pharmaceutical agents in AD, only four have been licensed: anticholinesterase inhibitors (donepezil, galantamine, and rivastigmine) that increase synaptic acetylcholine to aid learning and memory; and an NMDA-receptor antagonist (memantine). Interestingly, the neuroprotective effects of memantine were reported to be mediated by enhancing the astroglial system ${\text{X}}_{\text{c}}^{-}$ activity (Okada et al., 2019). The increased glutamate export appears to activate inhibitory metabotropic glutamate receptors to attenuate cognitive impairment from hyperactivation of thalamocortical glutamatergic transmission (Okada et al., 2019). However, the beneficial effects may result from inhibition of ferroptosis arising from the concomitant increased intracellular cystine/GSH (see above).

**Figure 1:**
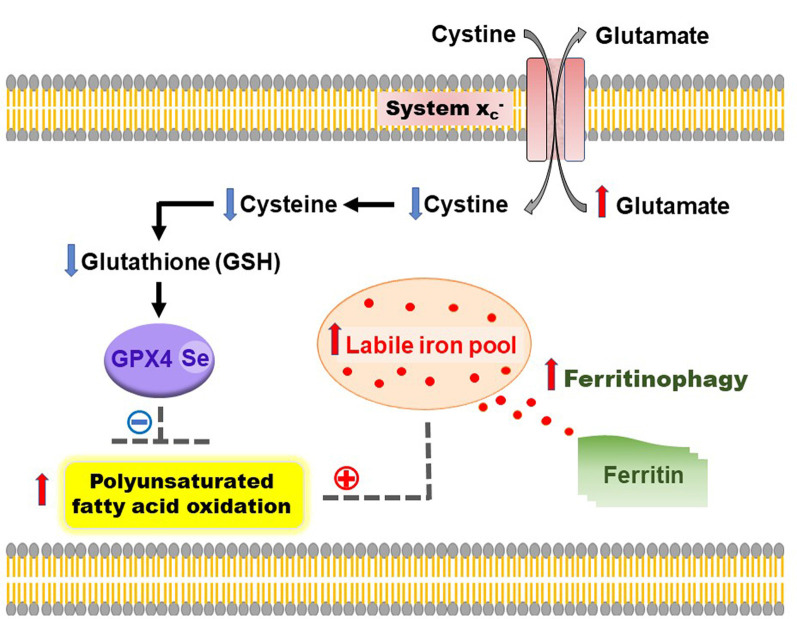
Overview of ferroptosis in Alzheimer’s disease (AD). During the process of ferroptosis, autophagy of ferritin (ferritinophagy) contributes to an increased labile iron pool, leading to elevated lipid peroxidation and oxidation of polyunsaturated fatty acids. This is aggravated by inhibition of the cystine/glutamate antiporter (system ${\text{X}}_{\text{c}}^{-}$), rendering the cell-deficient of cysteine, which has a domino effect of lowering glutathione (GSH) levels and impairs the activity of a selenium-dependent lipid repair enzyme, glutathione peroxidase-4 (GPX4) — a master regulator of ferroptosis. Combinatorial therapies targeted at maintaining iron homeostasis and restoring GSH levels/GPX4 will help to lower iron-induced lipid peroxidation and inhibit ferroptosis in AD.

Animals fed with excess iron demonstrate increased lipid peroxidation, BBB breakdown, altered mitochondrial dynamics, β-amyloid deposition, tau hyperphosphorylation, and loss of dendritic spine density—reminiscent of AD pathology (Sripetchwandee et al., 2014, 2016). Combinatorial therapy with deferiprone and N-acetylcysteine, exerted greater neuroprotection from iron-induced toxicity than monotherapy, including restored dendritic spine density, mitochondrial balance and ameliorated AD pathology (Sripetchwandee et al., 2014, 2016). Furthermore, the concept of targeting ferroptosis is supported by evidence of iron dyshomeostasis, altered system ${\text{X}}_{\text{c}}^{-}$ dynamics (diminished GSH/GPX4 activity) and enhanced lipid peroxidation in AD (Ashraf et al., 2020; Figure 1).

The challenges of conclusively identifying ferroptosis *in vivo* and post-mortem brain samples are hindered by the lack of specific markers for cells undergoing ferroptosis. It is important to state that different cell death patterns happen in parallel contributing to disease pathology. For example, it is known that ferroptosis and other types of cell deaths (necroptosis and apoptosis) occur concurrently following ischemic and traumatic brain injury (Zille et al., 2017; Magtanong and Dixon, 2018). The detection of markers that may indicate the presence of ferroptosis does not preclude the presence of other types of cell death. There are no established biomarkers that can detect ferroptosis in humans although different lines of evidence implicate a role for ferroptosis in AD. Development of a “ferroptosis-specific” antibody would be very informative in examining the effects of ferroptosis in multiple contexts including post-mortem samples and *in vivo* experiments.

Unlike clinical trials targeting β-amyloid, ferroptosis-modulating clinical trials have been exploratory and dose optimization still required as well as replication on a larger scale (Nikseresht et al., 2019). Concomitant identification of biomarkers for ferroptosis is also required for more rigorous inclusion/exclusion into clinical trials and robust evaluation/formal testing of novel therapies targeting the ferroptotic cascade. Many outstanding questions remain though—what are the individual contributions from microglia, astrocytes, oligodendrocytes, and neurons to ferroptosis? Is iron and its proteins differentially expressed in glia and neurons? What is the role of other transition metals, zinc, and copper, in ferroptosis and possible interactions with iron?

Mitochondria is the major site of energy production but coincidently for iron metabolism also. Mitochondrial dysfunction is thought to occur early in AD pathogenesis (Horowitz and Greenamyre, 2010). Damaged mitochondria are cleared by mitophagy to maintain mitochondrial homeostasis and shown to inhibit AD pathology in animal AD models (Fang et al., 2019). Furthermore, mitochondria depletion by Parkin-mediated mitophagy inhibited cysteine-deprivation induced ferroptosis (Basit et al., 2017). How iron metabolism relates to mitophagy, is mitophagy related to ferroptosis or a distinct phenomenon in AD remains to be addressed.

Neuroinflammation is a major characteristic of AD and represents a useful therapeutic target (Ong and Farooqui, 2005). Although crosstalk exists between neuroinflammation and iron metabolism (Urrutia et al., 2014), the relationship and contribution of ferroptosis to inflammation remains to be addressed.

Our discussion so far has focused on the cellular LIP, but 95% of functional iron in the body is in heme. Heme from hemoglobin breakdown can be a redox-active iron source, to induce/enhance lipid peroxidation and ferroptosis (NaveenKumar et al., 2018). AD is characterized by perturbed BBB permeability (Sripetchwandee et al., 2014, 2016). It will be pivotal to delineate the relationship between plasma and brain heme/iron homeostasis, and peripheral contributions to ferroptosis at different stages of the disease. Such knowledge will potentially identify peripheral ferroptosis biomarkers needed for future anti-ferroptosis trials to formally test ferroptosis contributions to AD and possibly other neurodegenerative diseases.

## Conclusion

Iron dyshomeostasis, impaired antioxidant defense, and lipid peroxidation are features of ferroptosis that could offer successful therapeutic targets in AD. Research on ferroptosis in the context of AD and other neurodegenerative diseases is still in its infancy. Exploration of the mechanism of ferroptosis and its role in AD has the potential to propose novel therapeutic approaches for, hitherto absent, highly effective treatments against AD and possibly, other neurodegenerative diseases.

## Author Contributions

AA conceived and designed the manuscript. P-WS reviewed and approved the manuscript and obtained the funding.

## Conflict of Interest

The authors declare that the research was conducted in the absence of any commercial or financial relationships that could be construed as a potential conflict of interest.
